# Supplementary material for: Peer groups for organisational learning: Clustering with practical constraints
Source: PLoS One. 2021 Jun 1;16(6):e0251723. doi: 10.1371/journal.pone.0251723 (PMC8168890; doi:10.1371/journal.pone.0251723)
Supplement: S1 Appendix — Complete descriptions of the steps in the three algorithms presented (kirigami-1, kirigami-2, and reallocation). (PDF) [file pone.0251723.s001.pdf]

# S1 Appendix

**Algorithm 1** Kirigami-1: Top-down constrained clustering for an upper cluster size threshold of $\lambda$.

Perform standard heirarchical clustering to get a series of partitions forming a tree: $\left\{\mathcal{P}^{(0)}, \mathcal{P}^{(1)}, ..., \mathcal{P}^{(n)}\right\}$, with $\mathcal{P}^{(0)} = \{\{1, ..., n\}\}$ and $\mathcal{P}^{(n)} = \{\{1\}, \{2\}, ..., \{n\}\}$.
For some goodness-of-fit index $G$, $t_{\max} := \mathrm{argmax}_t\big(G\big(\mathcal{P}^{(t)}\big)\big)$
$\mathcal{P} := \mathcal{P}^{(t_{\max})}$.
**while** $\exists P \in \mathcal{P}$ s.t. $|P| > \lambda$ **do**
    Find the smallest $t$ s.t. there exist a $P_p^{(t)}, P_q^{(t)} \in \mathcal{P}^{(t)}$ where $P = P_p^{(t)} \cup P_q^{(t)}$.
    $\mathcal{P} := (P / \{P\}) \cup \left\{P_p^{(t)}, P_q^{(t)}\right\}$.
**end while**
Return $\mathcal{P}$.

**Algorithm 2** Kirigami-2: Bottom-up constrained clustering for an upper cluster size threshold of $\lambda$. Dissimilarity function $d$ is defined by the type of heirarchical clustering.

Initialise the partition $\mathcal{P}^{(0)} := \{\{1\}, ..., \{n\}\}$, and dissimilarity matrix $D := d\big(\mathcal{P}^{(0)}\big)$.
$t := 0$.
**while** $\exists p, q$ s.t. $p \neq q$, $D_{p,q} < \infty$, and $\left|\mathcal{P}^{(t)}\right| > 1$ **do**
    $(p, q) := \mathrm{argmin}_{p,q} D_{p,q}$.
    **if** $\left|P_p^{(t)} \cup P_q^{(t)}\right| > \lambda$ **then**
        $D_{p,q} := \infty$.
    **else**
        $t := t + 1$
        $\mathcal{P}^{(t)} := \left(\mathcal{P}^{(t-1)} / \left\{P_p^{(t)}, P_q^{(t)}\right\}\right) \cup \left\{P_p^{(t)} \cup P_q^{(t)}\right\}$
        $D = d\big(\mathcal{P}^{(t)}\big)$
    **end if**
**end while**
For some goodness-of-fit index $G$, $t_{\max} := \mathrm{argmax}_t\big(G\big(\mathcal{P}^{(t)}\big)\big)$
Return $\mathcal{P}^{(t_{\max})}$.

**Algorithm 3** Reallocation: Hierarchical clustering based on a percentage $p \times 100\%$ of (potential) leavers and an upper cluster size threshold of $\lambda$. Number of observations is given by $N$. Set of old observations is given by $\mathcal{O}$, and set of new observations is given by $\mathcal{N}$. Dissimilarity function $d$ is defined by the type of heirarchical clustering. Distance metric for each observation $i$ to their assigned cluster is given by $\delta(i, \mathcal{P})$.

Allocate observations in $\mathcal{O}$ to based on previous year's partition $\mathcal{P}^{(0)} := \mathcal{P}^{\text{previous year}}$.
Let $O_L$ be the set of $\lfloor pN \rfloor$ observations with the highest $\delta(i, \mathcal{P})$ values.
$\mathcal{P}^{(0)} := \left\{P/O_L : P \in \mathcal{P}^{(0)}\right\} \cup \{\{i\} : i \in O_L\} \cup \{\{i\} : i \in \mathcal{N}\}$.
Compute dissimilarity matrix $D := d\big(\mathcal{P}^{(0)}\big)$.
$t := 0$.
**while** $\exists p, q$ s.t. $p \neq j$, $D_{p,q} < \infty$, and $\left|\mathcal{P}^{(t)}\right| > 1$ **do**
    $(p, q) := \text{argmin}_{p,q} D_{p,q}$.
    **if** $\left|P_p^{(t)} \cup P_q^{(t)}\right| > \lambda$ **then**
        $D_{p,q} := \infty$.
    **else**
        $t := t + 1$
        $\mathcal{P}^{(t)} := \left(\mathcal{P}^{(t-1)} / \left\{P_p^{(t)}, P_q^{(t)}\right\}\right) \cup \left\{P_p^{(t)} \cup P_q^{(t)}\right\}$
        $D = d\big(\mathcal{P}^{(t)}\big)$
    **end if**
**end while**
For some goodness-of-fit index $G$, $t_{\max} := \text{argmax}_t \big(G\big(\mathcal{P}^{(t)}\big)\big)$
Return $\mathcal{P}^{(t_{\max})}$.
