# Supplementary material for: Peer groups for organisational learning: Clustering with practical constraints
Source: PLoS One. 2021 Jun 1;16(6):e0251723. doi: 10.1371/journal.pone.0251723 (PMC8168890; doi:10.1371/journal.pone.0251723)
Supplement: S2 Appendix — Descriptions of linkage criteria and cluster goodness-of-fit indices used in this research. (PDF) [file pone.0251723.s002.pdf]

S2 Appendix

Table 1: Description of the four linkage criteria for heirarchical agglomerative clustering. The equation computes the dissimilarity between a cluster C_3 and the agglomeration of two other clusters C_1 and C_2. The dissimilarities between clusters are denoted by $D_{\cdot,\cdot}$ and the dissimilarities between single entities are denoted by $d_{\cdot,\cdot}$. Cluster sizes are given by n_1, n_2 and n_3.

Linkage Type	Description	Equation
single	Dissimilarity between two clusters is the minimum pairwise dissimilarity between one element from each cluster.	$\min(\{d_{i,j} : i \in C_1 \cup C_2\})$
complete	Dissimilarity between two clusters is the maximum pairwise dissimilarity between one element from each cluster.	$\max(\{d_{i,j} : i \in C_1 \cup C_2\})$
average	Dissimilarity between two clusters is the average pairwise dissimilarity between one element from each cluster, computed over all possible such pairs.	$\frac{\sum_{i \in C_1 \cup C_2, j \in C_3} d_{i,j}}{(n_1+n_2)n_3}$
Ward	When distance is Euclidean, dissimilarity is the increase in variance for the cluster being merged.	$\frac{n_1+n_3}{n_1+n_2+n_3} D_{1,3} + \frac{n_2+n_3}{n_1+n_2+n_3} D_{2,3} - \frac{n_3}{n_1+n_2+n_3} D_{1,2}$

Table 2: Descriptions of the three clustering criteria considered in this research. The numbers of observations and clusters are N and K respectively. The arithmetic mean of a given cluster k is given by $\bar{\mathbf{x}}_k$, and the overall mean is given by $\bar{\mathbf{x}}$.

Name	Description	Equation
Average Silhouette Width	The silhouette width is a measure defined for each observation i which compares the average distance between i and all other observations in the same cluster, $a(i)$, with the average distance between i and the closest other cluster, $b(i)$.<br>The silhouette width is calculated as<br>$$s(i) = (b(i) - a(i)) / \max(b(i), a(i)).$$<br>The average silhouette width is the arithmetic mean over the entire dataset.	$\frac{1}{N}\sum_{i=1}^{N} s(i)$
Calinski-Haribasz (CH) Index	Calculated as the ratio of the within-cluster variance and the between cluster variance, with a correction for "degrees of freedom". Highly similar to the F-statistic in Analysis-of-Variance.	$\frac{N-k}{k-1}\frac{\sum_{k=1}^{K}\sum_{i,j\in C_k, i\neq j} d(\mathbf{x}_i,\bar{\mathbf{x}}_k)^2}{\sum_{k=1}^{K} d(\bar{\mathbf{x}}_k,\bar{\mathbf{x}})^2}$
Pearson-Gamma	Equal to the Pearson correlation over each pair of observations i,j between the pairwise distance d_{ij} and the indicator $\mathrm{I}(i \sim j)$.	As described.
